# RESILIENCE AND POST-TRAUMATIC GROWTH: EXPLORING THE PERCEPTIONS OF GAINS AFTER ADVERSITY IN OLDER ADULTS

**DOI:** 10.1093/geroni/igz038.2639

**Published:** 2019-11-08

**Authors:** Lauren M Bouchard, Lydia K Manning

**Affiliations:** 1 Concordia University, River Forest, Illinois, United States; 2 Concordia University Chicago, River Forest, Illinois, United States

## Abstract

Resilience has been consistently shown across the literature as a protective factor in terms of aging successfully. Resilience is defined as a process of adjustment and adaptation, where painful life experiences can result in accumulative positive outcomes such as greater life meaning, hopefulness, and spiritual transcendence (Ramsey, 2012). These outcomes are also mentioned in the separate but related construct of “post-traumatic growth,” defined as positive outcomes (i.e. self-perception, improved interpersonal relationships, and a changed philosophy on life) which emerge after traumatic experiences (PTG; Tedschi & Calhoun, 1996). This study explored older adults perceptions on adaptation in regards to adverse life situations. Our findings indicate some participants were more likely to espouse resiliency and post-traumatic growth related explanations while others participants articulated difficulty in seeing the benefit related to the challenges they had faced. Similarly, participants faced a range of challenges from everyday stress to major life traumas, which also shaped perceptions of their own growth. Participants also indicated a range of orientations toward growth after adversity including denial, reluctance, acceptance, and optimism. Our results also suggest key differences in these constructs while they also remain similar and complementary in terms of our participants lives and stories. Our study also provides limitations and future directions in operationalizing PTG and resilience in the gerontological literature.

